# Identification of Potential Transcriptional Biomarkers Differently Expressed in Both* S. aureus*- and* E. coli*-Induced Sepsis via Integrated Analysis

**DOI:** 10.1155/2019/2487921

**Published:** 2019-04-09

**Authors:** Huan Chen, Ying Li, Tao Li, Hui Sun, Chuyi Tan, Min Gao, Wei Xing, Xianzhong Xiao

**Affiliations:** ^1^Department of Pathophysiology, School of Basic Medicine Science, Central South University, Changsha, China; ^2^Sepsis Translational Medicine Key Lab of Hunan Province, China; ^3^Department of Clinical Laboratory, Second Xiangya Hospital, Central South University, Changsha, China; ^4^Department of Intensive Medicine, Third Xiangya Hospital, Central South University, Changsha, China

## Abstract

Sepsis is a critical, complex medical condition, and the major causative pathogens of sepsis are both* Staphylococcus aureus* (*S. aureus*) and* Escherichia coli *(*E. coli*). Genome-wide studies identify differentially expressed genes for sepsis. However, the results for the identification of DEGs are inconsistent or discrepant among different studies because of heterogeneity of specimen sources, various data processing methods, or different backgrounds of the samples. To identify potential transcriptional biomarkers that are differently expressed in* S. aureus*- and* E. coli*-induced sepsis, we have analyzed four microarray datasets from GEO database and integrated results with bioinformatics tools. 42 and 54 DEGs were identified in both* S. aureus* and* E. coli* samples from any three different arrays, respectively. Hierarchical clustering revealed dramatic differences between control and sepsis samples. GO functional annotations suggested that DEGs in the* S. aureus* group were mainly involved in the responses of both defense and immune regulation, but DEGs in the* E. coli* group were mainly related to the regulation of endopeptidase activity involved in the apoptotic signaling pathway. Although KEGG showed inflammatory bowel disease in the E. coli group, the KEGG pathway analysis showed that these DEGs were mainly involved in the tumor necrosis factor signaling pathway, fructose metabolism, and mannose metabolism in both* S. aureus*- and* E. coli*-induced sepsis. Eight common genes were identified between sepsis patients with either* S. aureus* or* E. coli* infection and controls in this study. All the candidate genes were further validated to be differentially expressed by an ex-vivo human blood model, and the relative expression of these genes was performed by qPCR. The qPCR results suggest that GK and PFKFB3 might contribute to the progression of* S. aureus*-induced sepsis, and CEACAM1, TNFAIP6, PSTPIP2, SOCS3, and IL18RAP might be closely linked with* E. coli*-induced sepsis. These results provide new viewpoints for the pathogenesis of both sepsis and pathogen identification.

## 1. Introduction

Sepsis is the leading cause of death in noncoronary intensive care units, and sepsis has been increasing worldwide annually [1, 2]. Sepsis is a critical, complex medical condition, and is characterized as “a life-threatening organ dysfunction caused by a dysregulated host response to infection [3]”. The main causative pathogens of sepsis are bacteria, virus, and fungi, and* Staphylococcus aureus* (*S. aureus*) and* Escherichia coli* (*E. coli*) are common microorganisms detected in sepsis [4, 5]. Because every hour of delay after the first 6 hours increases mortality by 8% [6], both prompt diagnosis and treatment aid survival of sepsis.

In the last few decades, genome-wide studies identified candidate host genes for sepsis development, but only some of them classified the different pathophysiological mechanisms of sepsis caused by Gram-positive bacteria (or* S. aureus*) and Gram-negative bacteria (or* E. coli*) [7–13]. Tang et al. revealed 94 genes differentially expressed between intensive care unit patients with and without sepsis and the subgroups of gram-positive, gram-negative, and mixed infection samples had a similar transcriptional profile [14]. Ahn et al. identified classifier sets (human: two-factor and murine: four-factor) to distinguish* S. aureus* from healthy controls or* E. coli* bacteremia [12]. Thus, limitations still exist in any single study, and researchers wonder whether there are differently regulated genes among different types of microarrays. With an unbiased bioinformatics approach, we integrated the previous results and were able to discover effective and reliable biomarkers.

Therefore, the present study identifies significant host DEGs that are commonly regulated in* S. aureus*- and* E. coli*-induced sepsis by analyzing four microarray datasets from the gene expression omnibus (GEO) database. DEGs in* S. aureus*-induced sepsis vs. healthy controls or* E. coli*-induced sepsis vs. healthy controls were obtained by the R software (v3.3.2) and were enriched in any three datasets, and both the Gene Ontology (GO) process [15] and Kyoto Encyclopedia of Genes and Genomes (KEGG) pathways [16] were performed by the STRING database [17] and DAVID online tools [18, 19], respectively. Then, the DEGs screened from all four datasets were identified and validated in an ex-vivo model with quantitative real-time polymerase chain reaction (qPCR). Our study provided potential transcriptional biomarkers for sepsis diagnosis, as well as pathogen identification.

## 2. Methods

### 2.1. Identification of Eligible Microarray Datasets

GEO database is a public database supporting high-throughput gene expression data (www.ncbi.nlm.nih.gov/geo/). We searched GEO for relevant studies with key words “sepsis”, “homo sapiens”, “expression profiling by array”, “*Staphylococcus aureus*”, and “*Escherichia coli*”. A study was included in our analysis if the study fulfilled the following selection criteria: (1) study included both sepsis patients with positive culture results of* S. aureus* and* E. coli* and normal controls; (2) the sample was whole blood which is easier, available, and more widely used; (3) the study had almost whole genome-coverage (more than 10,000 genes) in each study. With these three selection criteria till January 1st, 2017, four datasets (GEO accession number numbers: GSE4607 [7–9], GSE25504 [10, 11], GSE33341 [12], and GSE65088 [13]) met the above inclusion criteria and were retained for subsequent analysis. The samples of these four datasets were from human blood. Within the four datasets, 9289 genes were extracted for subsequent analyses. Basic information of these datasets, such as published articles, patient characteristics, source of infection, and sampling time, is showed in Table 1.

### 2.2. Data Preprocessing and Screening for DEGs

All primary data of the four studies (GSE4607 [7–9], GSE25504 [10, 11], GSE33341 [12], and GSE65088 [13]) were downloaded from the GEO database and were analyzed, respectively, by R software and Bioconductor packages [20]. Firstly, arrays from Affymetrix were normalized with the “MAS5.0” normalization method [21], and Illumina arrays were normalized by GenomeStudio software (v2011.1, Illumina Inc.). Secondly, probe ID was converted into a unique official gene symbols; the symbol depended on the probe annotation information. Thirdly, to identify the DEGs within each dataset, we used the “limma” package [22] in R/Bioconductor to compare the gene expression between* S. aureus*/*E. coli* and control samples. Fourthly, the |log⁡2  Fold  Change| ≥1.5 and false discovery rate (FDR, Benjamini & Hochberg methods) < 0.05 were used as the cut-off values for screening DEGs. Fifthly, the DEGs were analyzed by the VennDiagram function [23] in R; this function identifies the genes common to* S. aureus*-/*E. coli*-induced sepsis. Finally, the hierarchical cluster analysis of the candidate DEGs in either* S. aureus* or* E. coli* samples vs. healthy controls was sorted with Cluster 3.0 software (Stanford University), and the results were visualized with TreeView Tool [24].

### 2.3. Functional Annotation Analyses

The common genes in any three datasets were collected to gain insight into the biological functions in GO enrichment analysis by the STRING database (https://string-db.org/cgi/input.pl). A* p*-value threshold of 0.05 was the statistically significant threshold, which identified significantly enriched GO biological process terms [15]. The KEGG database is a useful resource for pathway mapping, which integrate genomic, chemical, and systemic functional information [16]. DAVID is a group of online tools, which provide functional annotation of understanding biological meaning behind large list of genes (https://david.ncifcrf.gov/) [18, 19]. With DAVID, KEGG pathway enrichment analysis was conducted for the common genes with p < 0.05 considered statistically significant.

### 2.4. Quantitative Real-Time PCR

A human whole-blood model validated the candidate genes identified in the all four studies, and the expression of candidate genes was performed by qPCR. Anticoagulated blood sample of healthy human donors (n = 6, male, over the age of 18) was treated with both* S. aureus* ATCC 25923 (1 × 10^∧^7/mL) and the same dose of* E. coli* ATCC 25922; was incubated at 37°C with gentle rotation for 4 hours; and was treated with stroke-physiological-saline solution for mock infection. Both* S. aureus* and* E. coli* were inoculated with six different donors. Total RNA was collected from the incubated blood, was extracted with Blood Total RNA Rapid Extraction Kit (BioTeke, Beijing, China), and was reverse-transcribed with PrimeScript RT reagent Kit. All qPCR reaction mixtures were performed with SYBR Premix Ex Taq kit (TaKaRa, Dalian, China), and the primers used are listed in Table S1. We performed all the kits according to the manufacturers' instructions. Messenger RNA (mRNA) expression of all candidate genes was normalized to the expression of 18S rRNA, and the relative expression of gene transcript was calculated using the 2^−ΔΔCt^ method [25]. Human peripheral blood was collected from healthy volunteers after informed consent. The study was approved by the Ethics Committee of the Third Xiangya Hospital of Central South University, and written informed consent was obtained from all blood donors in accordance with the Declaration of Helsinki.

### 2.5. Statistical Analyses

All statistical analyses and graphs were performed with GraphPad Prism 7.00 software (GraphPad Software Inc.). Statistical differences among three groups were performed with one-way ANOVA followed by the Tukey test, and statistical differences are expressed as mean ± standard error of mean. All* p*-values are two-sided, and p < 0.05 was considered as statistically significant.

## 3. Results

### 3.1. Identification of DEGs and Common Genes across Four Datasets

**Figure 1:**
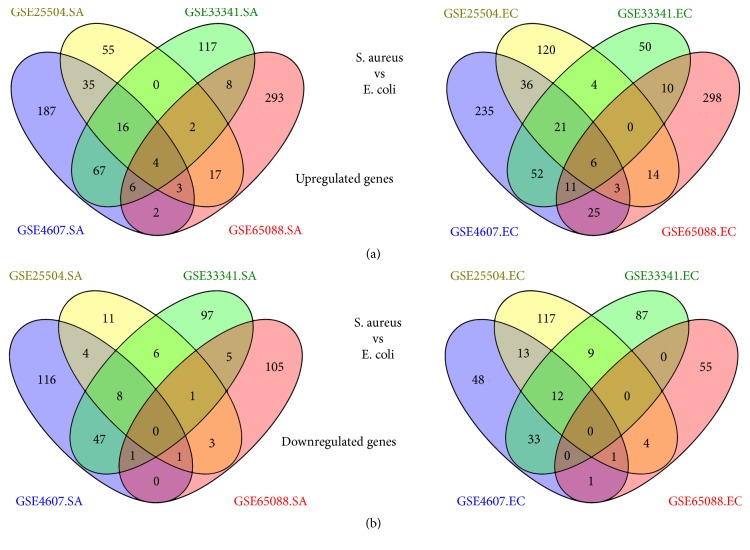
Summary of the differentially expressed genes in four candidate datasets. (a) Upregulated and (b) downregulated genes were screened out between* Staphylococcus aureus*- or* Escherichia coli*-induced sepsis and controls shown by Venn diagram. SA:* Staphylococcus aureus*; EC:* Escherichia coli*.

**Figure 2:**
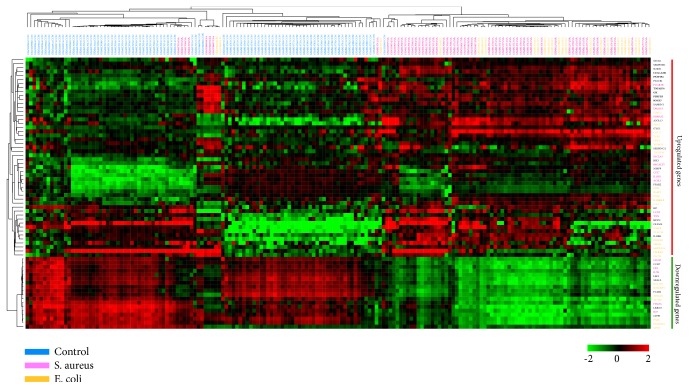
Hierarchical clustering of differentially expressed genes in* Staphylococcus aureus*,* Escherichia coli,* and control samples. Blue font for control samples, rose font for* Staphylococcus aureus* samples, and yellow font for* Escherichia coli* samples. Black font on the right side represents being expressed in both* Staphylococcus aureus* and* Escherichia coli* groups. Bold font on the right represents being screened in all four datasets.

With both |log⁡2  Fold  Change| ≥1.5 and FDR < 0.05 as cut-off criteria, we selected hundreds of significantly upregulated or downregulated genes in each dataset. Our selections are summarized in Tables S2 and S3. Among the candidates, 42 genes were selected repeatedly between* S. aureus*-induced sepsis patients and healthy controls in at least three of the four datasets. Among the 42 genes, 31 upregulated and 11 downregulated genes, shown in Figure 1(a) and Table S3, were selected. Yet there were 54 significantly common genes between* E. coli*-induced sepsis and normal controls. Among the 54 genes, 41 upregulated and 13 downregulated genes, shown in Figure 1(b) and Table S3, were screened. Hierarchical cluster analysis of these 96 selected genes revealed that remarkable differences existed between the control and sepsis samples, but a huge similarity was seen between* S. aureus* and* E. coli* groups (shown in Figure 2).

### 3.2. Functional Annotation Analysis

Functional enrichment analysis of the 42 or 54 common DEGs identified from any three datasets was performed separately in both* S. aureus*-induced sepsis and* E. coli*-induced sepsis, and the top 10 significantly enriched biological processes are listed in Table 2. In the GO analysis, “defense response” (GO: 0006952, p = 2.37E-04) was the most dramatically enriched function in sepsis caused by* S. aureus* (Table 2(a)), and “regulation of cysteine-type endopeptidase activity involved in apoptotic signaling pathway” (GO: 2001267, p = 2.61E-04) was the most highly enriched function in sepsis with* E. coli *infection (Table 2(b)). We used DAVID to analyze the total DEGs identified from any three studies, and the significantly enriched pathways of these genes were submitted to KEGG analysis. The results were shown in Table 3. The “tumor necrosis factor (TNF) signaling pathway” and the “fructose and mannose metabolism” were mainly enriched signaling pathways within the upregulated genes in both* S. aureus*- and* E. coli*-induced sepsis, while in the* E. coli *infection group “inflammatory bowel disease (IBD)” was an extra enrichment pathway within the upregulated genes. However, no significantly enriched pathway was identified in downregulated genes in either group.

### 3.3. Validation of the Most Commons across All Four Datasets by Ex-Vivo Experiments

To further investigate the common DEGs between* S. aureus*- and* E. coli*-induced sepsis, we screened eight key genes, which emerged in all four datasets. Surprisingly, none of common genes was found to be downregulated in either infection. Most notably, CEACAM1, GK, PFKFB3, and TNFAIP6 were increased in* S. aureus* group in four datasets, while CEACAM1, IL18RAP, LILRA5, PFKFB3, PSTPIP2, and SOCS3 were raised in* E. coli* group. The fold-change, T-test, and FDR-adjusted* p*-values of these eight key genes in the original four studied datasets are presented in Table 4.

**Figure 3:**
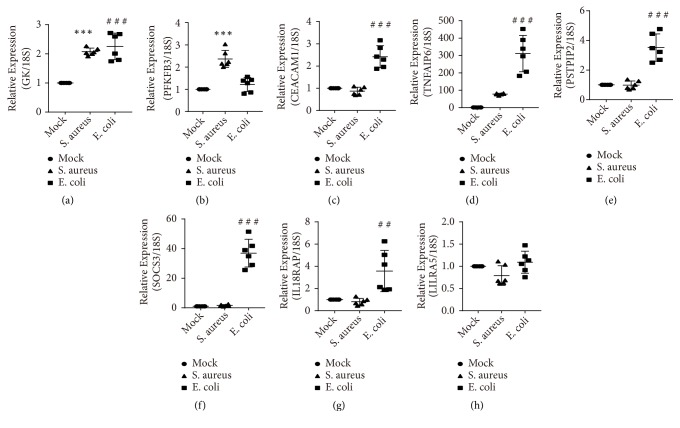
Quantitative real-time PCR results of eight common genes screened in all four datasets. (a) GK, (b) PFKFB3, (c) CEACAM1, (d) TNFAIP6, (e) PSTPIP2, (f) SOCS3, (g) IL18RAP, and (h) LILRA5 relative expression comparison between* Staphylococcus aureus*/* Escherichia coli* samples and controls. Expression of 18S rRNA was used as internal standard for normalization. *∗∗* represents* p* < 0.01, *∗∗∗* represents* p* < 0.001, when comparing* Staphylococcus aureus* group with mock infected group; ### represents* p* < 0.001, when comparing* Escherichia coli* group with mock infected group (one-way ANOVA followed by the Tukey test). CEACAM1: carcinoembryonic antigen related cell adhesion molecule 1; GK: glycerol kinase; PFKFB3: 6-phosphofructo-2-kinase/fructose-2, 6-biphosphatase 3; TNFAIP6: TNF alpha induced protein 6; IL18RAP: interleukin 18 receptor accessory protein; LILRA5: leukocyte immunoglobulin like receptor A5; PSTPIP2: proline-serine-threonine phosphatase interacting protein 2; SOCS3: suppressor of cytokine signaling 3.

To validate the eight candidate genes searched in all datasets, a human whole-blood ex-vivo model was carried out to detect the mRNA expression of these eight key genes by real-time qPCR. As shown in Figure 3, the expression levels of GK and PFKFB3 in the* S. aureus* group were higher than those of the mock infection group (p < 0.001, respectively, Figures 3(b), 3(c), and 3(d)). Also in Figure 3, GK, CEACAM1, TNFAIP6, PSTPIP2, SOCS3, and IL18RAP were remarkably higher in the* E. coli*-treated group than in the mock infection group (p < 0.001 and p < 0.01, respectively, Figures 3(a), 3(e), 3(f), and 3(g)). However, no significant difference existed in the LILRA5 level between the control and the* E. coli* group.

## 4. Discussion

Sepsis is one of the common causes of death in intensive care units [26–28]. The pathogenesis of sepsis involves invading pathogens, host immune responses, and multiple tissue damage caused by their complex interactions. Despite great progress made in understanding the pathophysiology of sepsis, we still lack indicators for early diagnosis. Therefore, the interaction between microorganisms and host is important to study, and understanding the molecular mechanisms of sepsis development is important.

Microarray studies that detect the mRNA levels of millions of genes in human beings provide an opportunity for early diagnosis in sepsis [29]. Because only a few identified the changes of host expression levels in different pathogen infections in sepsis, clear and effective diagnostic biomarkers are unknown. Most studies came from either a single cohort study or a multiple pathogen background. In addition, the results for the identification of DEGs are inconsistent or discrepant among different studies because of heterogeneity of specimen sources (e.g., blood, peripheral blood mononuclear cells, and neutrophils), diverse types of pathogens, various data processing methods, or different backgrounds of the samples. Therefore, confounding effects cannot be eliminated in these studies.

In this study, we identified, with R software, DEGs with both* S. aureus*- and* E. coli*-induced sepsis in four different gene expression profiling datasets, and integrated common DEGs for deep analyses by informatics tools. Based on four public GEO datasets with case-control study design, we identified 42 notable genes with* S. aureus* samples (31 upregulated and 11 downregulated) and 54 significantly changed genes with* E. coli* patients (41 upregulated and 13 downregulated). Both the 42 genes and the 54 genes are commonly regulated in at least three different arrays, respectively.

The microarray and the pathophysiology of sepsis are consistent. From hierarchical clustering analysis, remarkable differences between control and sepsis samples were observed, but, unfortunately, many similarities between* S. aureus*- and* E. coli*-induced sepsis were observed. These results were essentially in agreement with previous studies. For instance, Tang confirmed that sepsis patients with Gram-positive and Gram-negative infection had a homogeneous host response at the transcriptional level [14]. In fact, the clinical features of Gram-positive and Gram-negative sepsis are not easily distinguishable [30]. It is usually thought that this conservative program of gene expression might be part of host's general “alarm signal” to maximize the detection of invasive pathogens.

Nevertheless, the heterogeneity of the pathogenic mechanism remained in two bacterial infections; this observation was seen in both the GO analyses and the KEGG pathway enrichments of DEGs. DEGs were analyzed by GO functional annotation, which showed that DEGs in* S. aureus* group were mainly involved in the responses of both defense and immune regulation; however, common genes of* E. coli* group were mainly related to the regulation of endopeptidase activity involved in the apoptotic signaling pathway. Furthermore, the enriched KEGG pathways of common genes in* S. aureus*-induced sepsis included both the TNF signaling pathway and fructose and mannose metabolic pathway, while the KEGG pathway enrichments in sepsis with* E. coli* infection consisted of TNF signaling pathway, IBD, and fructose and mannose metabolism. TNF signaling pathway is intimately implicated in the innate immune response in the development of sepsis [31]. As one of the most important proinflammatory cytokines, TNF-*α* can mediate a wide range of pathways such as both apoptosis and inflammation [32] and has been defined as a major component in the pathogenesis of sepsis [33]. One experimental mouse model suggested that the deficiency of the TNF receptor I could protect mice from both lipopolysaccharides (LPS) and* S. aureus*-enterotoxin B induced septic shock [34]. Fructose and mannose metabolism leads to enhanced glycolysis and N-glycan biosynthesis [35, 36], anaerobic glycolysis may be a novel therapeutic target for sepsis-related acute lung injury [35], and the product lactose is closely bound up with septic shock [3]. Therefore, these two pathways may play important roles in the development of sepsis induced by* S. aureus *and* E. coli* and may provide potential insights of the therapeutic strategies in sepsis.

As for the eight common genes screened out in all four datasets, CEACAM1, GK, PFKFB3, and TNFAIP6 emerged repeatedly in the* S. aureus* group, but CEACAM1, IL18RAP, LILRA5, PFKFB3, PSTPIP2, and SOCS3 emerged in the* E. coli* infection. Both CEACAM1 and PFKFB3 were reduplicated. Then, by detecting the changes of mRNA expression, we validated these eight key genes in an ex-vivo experiment of both* S. aureus*- and* E. coli*-treated human whole-blood samples.

Importantly, we revealed that GK and PFKFB3 were upregulated in* S. aureus* group, yet GK, CEACAM1, TNFAIP6, PSTPIP2, SOCS3, and IL18RAP were increased in* E. coli* group. The protein encoded by PFKFB3 is an important enzyme in glycolysis, and the protein contributes to cell apoptosis, enhancement of ROS, and the development of sepsis [35–37]. GK is a key enzyme in the regulation of glycerol uptake and metabolism, and a study found that GK was increased in the septic rat models [38]. TNFAIP6 is upregulated in response to many proinflammatory cytokines such as TNF-*α* and interleukin-1, and elevated levels of TNFAIP6 have been reported in the plasma of both LPS stimulation [39] and* S. aureus*-induced mastitis [40]. CEACAM1 is a receptor on neutrophils, and CEACAM1 negatively regulates both NLRP3 inflammasome activation and immune response [41–43] and has been found to increase the susceptibility of bacterial infection [44]. PSTPIP2 is an actin-associated protein expressed in macrophages, and PSTPIP2 regulates both filopodia formation and directional motility of the macrophage [45]. SOCS3 plays important role in the course of sepsis and is reportedly involved in the proinflammatory phenotype polarization of the M1 macrophage [46, 47]. IL18RAP is a subunit of the heterodimeric receptor for interleukin 18 and is reported to be elevated in* E. coli*-caused bacteremia [48]. A mutation of IL18RAP is closely related to both Crohn's disease and IBD [49–51]. LILRA5 is involved in both macrophage activation and secretion of several proinflammatory cytokines, and LILRA5 has a potential impact on pathogenesis of rheumatoid arthritis [52]. However, the expression of LILRA5 mRNA has no difference between* E. coli* infection and control group by qPCR in the ex-vivo model.

In this study, qPCR results further indicated that almost of eight candidate genes were expressed differently in different bacterial infections, and qPCR has the potential to distinguish* S. aureus* and* E. coli *infections. Our qPCR conclusion roughly agrees with studies previously reported. Although the exact contributions of these genes to identify both* S. aureus*- and* E. coli*-induced sepsis are not clear yet, further research should investigate these eight genes as potential transcriptional biomarkers for pathogen identification in sepsis. Hence, to achieve a more convincible conclusion, further validation using patient samples is as well required.

In conclusion, we identified 42 or 53 DEGs that were differentially expressed between sepsis patients with* S. aureus* or* E. coli* infection and healthy controls, respectively. GO and pathway enrichment analysis revealed that these common markers were strongly associated with immune response or regulation of endopeptidase activity. The qPCR results suggested that GK and PFKFB3 might contribute to the progression of* S. aureus*-induced sepsis, and GK, CEACAM1, TNFAIP6, PSTPIP2, SOCS3, and IL18RAP might be closely linked with* E. coli*-induced sepsis. Our study has gained novel insight into sepsis pathogenesis and has confirmed systematic changes in different gene expression patterns between* S. aureus*- and* E. coli*-induced sepsis. Such insights may ultimately lead to early pathogen identification in sepsis.

## Figures and Tables

**Table 1:** Summary of representative clinical studies comparing the host transcriptomic responses in *Staphylococcus aureus*-/*Escherichia coli*-induced sepsis.

Published article	Dataset accession number;*∗* platform	Clinical setting	Cell type	Patient group	Control group	Microbiological cause	Infection source	Sampling time
Wong HR, et al (2007, 2012) [7, 8]; Cvijanovich N, et al (2008) [9]	GSE4607; GPL570	PICU	Whole blood	Septic shock(n=42)	Outpatients or inpatients without infective pathology(n=15)	*S. aureus*(n=9); *E. coli*(n=3)	Lung, blood, urinary tract, colitis, CNS, abdominal, soft tissue, unknown	1-3 days
Smith CL, et al (2014) [10]; Dickinson P (2015) [11]	GSE25504; GPL6947	Neonatal unit	Whole blood	Sepsis(n=26)	Inpatients without infective pathology(n=35)	*S. aureus*(n=18); *E. coli*(n=1)	Lung, CNS, urinary tract, abdominal, soft tissue, unspecified	Within 6 hours
Ahn SH, et al (2013) [12]	GSE33341; GPL571	Adult inpatient	Whole blood	Sepsis with positive blood cultures(n=51)	Healthy volunteers(n=43)	*S. aureus*(n=32); *E. coli*(n=19)	Lung, urinary tract, endocarditis, skin, catheter, bone, CNS, unknown	1day
Dix A, et al (2015) [13]	GSE65088; GPL10558	none	Whole blood	Anti-coagulated blood of healthy human donors was incubated with bacteria or fungi(n=36)	Anti-coagulated blood of healthy human donors was incubated with mock-infected control(n=20)	*S. aureus*(n=3); *E. coli*(n=4)	blood	8 hours

*Note*. PICU: pediatric intensive care unit; CNS: central nervous system.

*∗*GEO Datasets information from PubMed (https://www.ncbi.nlm.nih.gov/geo).

**Table:** (a) *Staphylococcus aureus*

GO Term (Biological Process)	Function description	Gene count	*P*-value	Gene symbol
GO.0006952	defense response	15	2.37E-04	ANXA3, CCR7, CD247, FAIM3, FCGR1B, FFAR2, HP, IL1RN, LCN2, PLSCR1, S100A12, SERPING1, SOCS3, TNFAIP6, TXN
GO.0006955	immune response	14	4.23E-04	ANXA3, CCR7, CD247, CD96, CST7, FCGR1B, FFAR2, IL7R, LCN2, LEF1, S100A12, SERPING1, SOCS3, TXN
GO.0002376	immune system process	16	7.81E-04	ANXA3, CCR7, CD247, CD96, CEACAM1, CST7, FAIM3 FCGR1B, FFAR2, HP, LCN2, PLSCR1, S100A12, SERPING1, SOCS3, TXN
GO.0050776	regulation of immune response	11	7.81E-04	CCR7, CD247, CD96, FCGR1B, FFAR2, IL7R, OLFM4, PLSCR1, SAMSN1, SERPING1, SOCS3
GO.0002682	regulation of immune system process	13	1.87E-03	CCR7, CD247, CD96, FCGR1B, FFAR2, IL7R, LEF1, OLFM4, PLSCR1, SAMSN1, SERPING1, SOCS3, STOM
GO.0048583	regulation of response to stimulus	19	4.55E-03	CCR7, CD247, CD96, FCGR1B, FFAR2, HP, IL18R1, IL1RN, IL7R, LEF1, MMP9, OLFM4, PLSCR1, S100A12, SAMSN1, SERPING1, STOM, TNFAIP6, TXN
GO.0065009	regulation of molecular function	17	4.68E-03	ACSL1, ANXA3, CCR7, CST7, HP, ID3, IL1RN, LEF1, MMP9, PLSCR1, S100A12, SERPINB1, SERPING1, SOCS3, SORT1, STOM, TXN
GO.0044092	negative regulation of molecular function	11	6.64E-03	ANXA3, CST7, HP, ID3, IL1RN, LEF1, MMP9, SERPINB1, SERPING1, SOCS3, SORT1
GO.0050790	regulation of catalytic activity	15	8.01E-03	ACSL1, ANXA3, CCR7, CST7, HP, IL1RN, LEF1, MMP9, PLSCR1, S100A12, SERPINB1, SERPING1, SOCS3, SORT1, TXN
GO.0071345	cellular response to cytokine stimulus	8	8.51E-03	ACSL1, CCR7, FCGR1B, IL18R1, IL1RN, IL7R, LEF1, SOCS3

**Table:** (b) *Escherichia coli*

GO Term (Biological Process)	Function description	Gene count	*P*-value	Gene symbol
GO.2001267	regulation of cysteine-type endopeptidase activity involved in apoptotic signaling pathway	5	2.61E-04	JAK2, MMP9, TNFRSF10A, TNFRSF10B, TNFSF10
GO.0010951	negative regulation of endopeptidase activity	8	1.20E-03	LEF1, MMP9, SERPINB1, SERPING1, TIMP1, TNFRSF10A, TNFRSF10B, TNFSF10
GO.0097296	activation of cysteine-type endopeptidase activity involved in apoptotic signaling pathway	4	1.20E-03	JAK2, TNFRSF10A, TNFRSF10B, TNFSF10
GO.0052548	regulation of endopeptidase activity	9	1.82E-03	JAK2, LEF1, MMP9, SERPINB1, SERPING1, TIMP1, TNFRSF10A, TNFRSF10B, TNFSF10
GO.0051346	negative regulation of hydrolase activity	9	2.24E-03	LEF1, MMP9, SERPINB1, SERPING1, SORT1, TIMP1, TNFRSF10A, TNFRSF10B, TNFSF10
GO.0043086	negative regulation of catalytic activity	12	2.44E-03	ANXA3, HP, LEF1, MMP9, SERPINB1, SERPING1, SOCS3, SORT1, TIMP1, TNFRSF10A, TNFRSF10B, TNFSF10
GO.0050790	regulation of catalytic activity	19	2.52E-03	ANXA3, CCR7, FPR1, HP, JAK2, LEF1, MMP9, PFKFB2, PLSCR1, RASGRP1, SERPINB1, SERPING1, SOCS3, SORT1, TIMP1, TNFRSF10A, TNFRSF10B, TNFSF10, VPS9D1
GO.0006954	inflammatory response	9	2.59E-03	CCR7, FFAR2, HP, IL18RAP, ORM1, PLSCR1, RASGRP1, TLR5, TNFAIP6
GO.0009605	response to external stimulus	17	4.17E-03	ABLIM1, ANXA3, ARG1, AUTS2, BCL11B, CCR7, FFAR2, FPR1, HP, JAK2, LEF1, MMP9, PLSCR1, SOCS3, TNFRSF10A, TNFRSF10B, UPP1
GO.0044092	negative regulation of molecular function	13	4.17E-03	ANXA3, HP, JAK2, LEF1, MMP9, SERPINB1, SERPING1, SOCS3, SORT1, TIMP1, TNFRSF10A, TNFRSF10B, TNFSF10

**Table:** (a) *Staphylococcus aureus*

KEGG Term	Function description	Gene count	*P*-value	Gene symbol
ptr04668	TNF signaling pathway	3	1.17E-02	IL18R1, SOCS3, MMP9
ptr00051	Fructose and mannose metabolism	2	4.93E-02	PFKFB3, HK3

**Table:** (b) *Escherichia coli*

KEGG Term	Function description	Gene count	*P*-value	Gene symbol
ptr00051	Fructose and mannose metabolism	3	4.05E-03	PFKFB3, HK3, PFKFB2
ptr05321	Inflammatory bowel disease (IBD)	3	1.65E-02	IL18R1, IL18RAP, TLR5
ptr04668	TNF signaling pathway	3	4.05E-02	IL18R1, SOCS3, MMP9

*Note*. KEGG: Kyoto Encyclopedia of Genes and Genomes; TNF: tumor necrosis factor.

**Table:** (a) *Staphylococcus aureus*

Gene symbol	GSE4607			GSE25504			GSE33341			GSE65088		
	FC	*P*-value		FC	*P*-value		FC	*P*-value		FC	*P*-value	
		T-Test	FDR		T-Test	FDR		T-Test	FDR		T-Test	FDR
CEACAM1	2.44	3.62E-07	3.54E-05	2.42	3.00E-10	1.03E-08	2.72	2.11E-21	9.92E-20	2.36	9.14E-05	9.33E-03
GK	1.64	2.11E-09	9.94E-07	1.61	2.51E-10	9.01E-09	1.77	1.11E-18	2.81E-17	3.11	8.00E-07	7.88E-03
PFKFB3	2.54	7.83E-09	2.49E-06	1.87	5.36E-12	4.25E-10	2.13	5.85E-21	2.43E-19	1.89	7.01E-05	8.15E-03
TNFAIP6	1.77	1.43E-04	2.94E-03	2.53	7.09E-11	3.25E-09	3.18	3.06E-20	1.07E-18	1.97	9.19E-04	3.55E-02

**Table:** (b) *Escherichia coli*

Gene symbol	GSE4607			GSE25504			GSE33341			GSE65088		
	FC	P-value		FC	P-value		FC	P-value		FC	P-value	
		T-Test	FDR		T-Test	FDR		T-Test	FDR		T-Test	FDR
CEACAM1	2.83	2.61E-07	1.44E-04	2.79	2.67E-03	3.90E-02	2.77	2.33E-18	1.01E-16	1.63	3.54E-05	3.57E-03
IL18RAP	2.42	2.40E-05	2.48E-03	2.65	8.07E-04	1.72E-02	2.27	3.74E-19	2.01E-17	2.06	8.72E-04	2.77E-02
LILRA5	2.27	1.16E-03	3.08E-02	2.32	2.02E-04	6.20E-03	2.84	7.28E-16	1.64E-14	2.58	3.50E-07	2.21E-04
PFKFB3	2.23	8.13E-06	1.32E-03	3.28	5.94E-11	2.34E-08	1.93	1.31E-16	3.57E-15	2.61	4.82E-06	1.03E-03
PSTPIP2	2.77	1.03E-08	1.82E-05	1.92	2.11E-07	2.37E-05	2.34	2.17E-24	7.80E-22	1.93	1.23E-03	3.46E-02
SOCS3	2.43	1.07E-04	6.73E-03	1.99	1.93E-14	2.29E-11	1.79	1.76E-13	2.38E-12	3.64	3.05E-07	2.10E-04

*Note*. FC: fold-change; FDR: false discovery rate; CEACAM1: carcinoembryonic antigen related cell adhesion molecule 1; GK: glycerol kinase; PFKFB3: 6-phosphofructo-2-kinase/fructose-2, 6-biphosphatase 3; TNFAIP6: TNF alpha induced protein 6; IL18RAP: interleukin 18 receptor accessory protein; LILRA5: leukocyte immunoglobulin like receptor A5; PSTPIP2: proline-serine-threonine phosphatase interacting protein 2; SOCS3: suppressor of cytokine signaling 3.

## Data Availability

The datasets supporting the conclusions of this article are within the article and its additional files.

## Abbreviations

CEACAM1: Carcinoembryonic antigen related cell adhesion molecule 1
CNS: Central nervous system
DEGs: Differentially expressed genes
*E. coli*: * Escherichia coli*
FC: Fold-change
FDR: False discovery rate
GEO: Gene expression omnibus
GK: Glycerol kinase
GO: Gene ontology
IBD: Inflammatory bowel disease
IL18RAP: Interleukin 18 receptor accessory protein
KEGG: Kyoto Encyclopedia of Genes and Genomes
LILRA5: Leukocyte immunoglobulin like receptor A5
LPS: Lipopolysaccharides
mRNA: Messenger RNA
PFKFB3: 6-Phosphofructo-2-kinase/fructose-2, 6-biphosphatase 3
PICU: Pediatric intensive care unit
PSTPIP2: Proline-serine-threonine phosphatase interacting protein 2
qPCR: Quantitative real-time polymerase chain reaction
*S. aureus*: * Staphylococcus aureus*
SOCS3: Suppressor of cytokine signaling 3
TNF: Tumor necrosis factor
TNFAIP6: TNF alpha induced protein 6.

## Supplementary Materials

Supplementary Materials Supplementary Table 1 Primers designed for validation of commonly expressed genes in four datasets by quantitative real-time PCR. Supplementary Table 2 is a summary of DEGs selected from each dataset using |log2 Fold Change| ≥1.5 and FDR < 0.05 as cut-off criteria. Supplementary Table 3 is the common genes in any three datasets.

## References

[1] Vincent J.-L., Marshall J. C., Ñamendys-Silva S. A. (2014). Assessment of the worldwide burden of critical illness: the Intensive Care Over Nations (ICON) audit. *The Lancet Respiratory Medicine*.

[2] Seymour C. W., Gesten F., Prescott H. C. (2017). Time to treatment and mortality during mandated emergency care for sepsis. *The New England Journal of Medicine*.

[3] Singer M., Deutschman C. S., Seymour C. (2016). The third international consensus definitions for sepsis and septic shock (sepsis-3). *Journal of the American Medical Association*.

[4] Glik J., Kawecki M., Gaździk T., Nowak M. (2012). The impact of the types of microorganisms isolated from blood and wounds on the results of treatment in burn patients with sepsis. *Polish Journal of Surgery*.

[5] Faix J. D. (2013). Biomarkers of sepsis. *Critical Reviews in Clinical Laboratory Sciences*.

[6] Kumar A., Roberts D., Wood K. E. (2006). Duration of hypotension before initiation of effective antimicrobial therapy is the critical determinant of survival in human septic shock. *Critical Care Medicine*.

[7] Wong H. R., Shanley T. P., Sakthivel B. (2007). Genome-level expression profiles in pediatric septic shock indicate a role for altered zinc homeostasis in poor outcome. *Physiological Genomics*.

[8] Wong H. R., Cvijanovich N. Z., Hall M. (2012). Interleukin-27 is a novel candidate diagnostic biomarker for bacterial infection in critically ill children. *Critical Care*.

[9] Cvijanovich N., Shanley T. P., Lin R. (2008). Validating the genomic signature of pediatric septic shock. *Physiological Genomics*.

[10] Smith C. L., Dickinson P., Forster T. (2014). Identification of a human neonatal immune-metabolic network associated with bacterial infection. *Nature Communications*.

[11] Dickinson P., Smith C. L., Forster T. (2015). Whole blood gene expression profiling of neonates with confirmed bacterial sepsis. *Genomics Data*.

[12] Ahn S. H., Tsalik E. L., Cyr D. D. (2013). Gene expression-based classifiers identify staphylococcus aureus infection in mice and humans. *PLoS ONE*.

[13] Dix A., Hunniger K., Weber M., Guthke R., Kurzai O., Linde J. (2015). Biomarker-based classification of bacterial and fungal whole-blood infections in a genome-wide expression study. *Frontiers in Microbiology*.

[14] Tang B. M. P., McLean A. S., Dawes I. W., Huang S. J., Cowley M. J., Lin R. C. Y. (2008). Gene-expression profiling of Gram-positive and Gram-negative sepsis in critically ill patients. *Critical Care Medicine*.

[15] Oliveira G. S., Santos A. R. (2016). Using the Gene Ontology tool to produce de novo protein-protein interaction networks with IS_A relationship. *Genetics and Molecular Research*.

[16] Kanehisa M., Furumichi M., Tanabe M., Sato Y., Morishima K. (2017). KEGG: new perspectives on genomes, pathways, diseases and drugs. *Nucleic Acids Research*.

[17] Szklarczyk D., Morris J. H., Cook H. (2017). The STRING database in 2017: quality-controlled protein-protein association networks, made broadly accessible. *Nucleic Acids Research*.

[18] Huang D. W., Sherman B. T., Lempicki R. A. (2009). Systematic and integrative analysis of large gene lists using DAVID bioinformatics resources. *Nature Protocols*.

[19] Huang D. W., Sherman B. T., Lempicki R. A. (2009). Bioinformatics enrichment tools: paths toward the comprehensive functional analysis of large gene lists. *Nucleic Acids Research*.

[20] Gentleman R. C., Carey V. J., Bates D. M. (2004). Bioconductor: open software development for computational biology and bioinformatics. *Genome Biology*.

[21] Pepper S. D., Saunders E. K., Edwards L. E., Wilson C. L., Miller C. J. (2007). The utility of MAS5 expression summary and detection call algorithms. *BMC Bioinformatics*.

[22] Ritchie M. E., Phipson B., Wu D. (2015). *limma* powers differential expression analyses for RNA-sequencing and microarray studies. *Nucleic Acids Research*.

[23] Chen H., Boutros P. C. (2011). VennDiagram: A package for the generation of highly-customizable Venn and Euler diagrams in R. *BMC Bioinformatics*.

[24] Saldanha A. J. (2004). Java Treeview--extensible visualization of microarray data. *Bioinformatics*.

[25] Arocho A., Chen B., Ladanyi M., Pan Q. (2006). Validation of the 2-DeltaDeltaCt calculation as an alternate method of data analysis for quantitative PCR of BCR-ABL P210 transcripts. *Diagnostic Molecular Pathology*.

[26] Dokter J., Felix M., Krijnen P. (2015). Mortality and causes of death of Dutch burn patients during the period 2006-2011. *Burns*.

[27] Moskowitz A., Omar Y., Chase M. (2017). Reasons for death in patients with sepsis and septic shock. *Journal of Critical Care*.

[28] West T. E., Wikraiphat C., Tandhavanant S. (2017). Patient characteristics, management, and predictors of outcome from severe community-onset staphylococcal sepsis in northeast thailand: a prospective multicenter study. *The American Journal of Tropical Medicine and Hygiene*.

[29] Li Y., Li Y., Bai Z., Pan J., Wang J., Fang F. (2017). Identification of potential transcriptomic markers in developing pediatric sepsis: a weighted gene co-expression network analysis and a case-control validation study. *Journal of Translational Medicine*.

[30] Jenner R. G., Young R. A. (2005). Insights into host responses against pathogens from transcriptional profiling. *Nature Reviews Microbiology*.

[31] Matsukawa A., Kaplan M. H., Hogaboam C. M., Lukacs N. W., Kunkel S. L. (2001). Pivotal role of signal transducer and activator of transcription (Stat)4 and Stat6 in the innate immune response during sepsis. *The Journal of Experimental Medicine*.

[32] Lin W.-J., Yeh W.-C. (2005). Implication of Toll-like receptor and tumor necrosis factor *α* signaling in septic shock. *Shock*.

[33] Hehlgans T., Pfeffer K. (2005). The intriguing biology of the tumour necrosis factor/tumour necrosis factor receptor superfamily: players, rules and the games. *The Journal of Immunology*.

[34] Pfeffer K., Matsuyama T., Kündig T. M. (1993). Mice deficient for the 55 kd tumor necrosis factor receptor are resistant to endotoxic shock, yet succumb to * L. monocytogenes* infection. *Cell*.

[35] Gong Y., Lan H., Yu Z. (2017). Blockage of glycolysis by targeting PFKFB3 alleviates sepsis-related acute lung injury via suppressing inflammation and apoptosis of alveolar epithelial cells. *Biochemical and Biophysical Research Communications*.

[36] Li F., Liu J., Bao R. (2018). Acetylation accumulates PFKFB3 in cytoplasm to promote glycolysis and protects cells from cisplatin-induced apoptosis. *Nature Communications*.

[37] L'Her E., Sebert P. (2001). A global approach to energy metabolism in an experimental model of sepsis. *American Journal of Respiratory and Critical Care Medicine*.

[38] Chang C. K., Moskal S. F., Srivenugopal K. S., Schumer W. (1993). Altered levels of mRNA encoding enzymes of hepatic glucose metabolism in septic rats. *Circulatory Shock*.

[39] Allantaz-Frager F., Turrel-Davin F., Venet F. (2013). Identification of biomarkers of response to IFNg during endotoxin tolerance: application to septic shock. *PLoS ONE*.

[40] Cremonesi P., Capoferri R., Pisoni G. (2012). Response of the goat mammary gland to infection with Staphylococcus aureus revealed by gene expression profiling in milk somatic and white blood cells. *BMC Genomics*.

[41] Lee H. S. W., Ostrowski M. A., Gray-Owen S. D. (2008). CEACAM1 dynamics during Neisseria gonorrhoeae suppression of CD4 + T lymphocyte activation. *The Journal of Immunology*.

[42] Lu R., Pan H., Shively J. E. (2012). CEACAM1 negatively regulates IL-1*β* production in LPS activated neutrophils by recruiting SHP-1 to a SYK-TLR4-CEACAM1 complex. *PLoS Pathogens*.

[43] Rowe H. A., Griffiths N. J., Hill D. J., Virji M. (2007). Co-ordinate action of bacterial adhesins and human carcinoembryonic antigen receptors in enhanced cellular invasion by capsulate serum resistant *Neisseria meningitidis*. *Cellular Microbiology*.

[44] Griffiths N. J., Bradley C. J., Heyderman R. S., Virji M. (2007). IFN-*γ* amplifies NF*κ*B-dependent Neisseria meningitidis invasion of epithelial cells via specific upregulation of CEA-related cell adhesion molecule 1. *Cellular Microbiology*.

[45] Chitu V., Pixley F. J., Macaluso F. (2005). The PCH family member MAYP/PSTPIP2 directly regulates F-actin bundling and enhances filopodia formation and motility in macrophages. *Molecular Biology of the Cell (MBoC)*.

[46] Qin H., Holdbrooks A. T., Liu Y., Reynolds S. L., Yanagisawa L. L., Benveniste E. N. (2012). SOCS3 deficiency promotes M1 macrophage polarization and inflammation. *The Journal of Immunology*.

[47] Lv R., Zhao J., Lei M., Xiao D., Yu Y., Xie J. (2017). IL-33 attenuates sepsis by inhibiting Il-17 receptor signaling through upregulation of SOCS3. *Cellular Physiology and Biochemistry*.

[48] Yu J.-Y., Zhang B., Peng L. (2015). Repositioning of memantine as a potential novel therapeutic agent against meningitic * E. coli*-induced pathogenicities through disease-associated alpha7 cholinergic pathway and RNA sequencing-based transcriptome analysis of host inflammatory responses. *PLoS ONE*.

[49] Andiappan A. K., Melchiotti R., Poh T. Y. (2015). Genome-wide analysis of the genetic regulation of gene expression in human neutrophils. *Nature Communications*.

[50] Harrison O. J., Srinivasan N., Pott J. (2015). Epithelial-derived IL-18 regulates Th17 cell differentiation and Foxp3(+) Treg cell function in the intestine. *Mucosal Immunology*.

[51] Liu H., Irwanto A., Tian H. (2012). Identification of IL18RAP/IL18R1 and IL12B as leprosy risk genes demonstrates shared pathogenesis between inflammation and infectious diseases. *American Journal of Human Genetics*.

[52] Mitchell A., Rentero C., Endoh Y. (2008). LILRA5 is expressed by synovial tissue macrophages in rheumatoid arthritis, selectively induces pro-inflammatory cytokines and IL-10 and is regulated by TNF-*α*, IL-10 and IFN-*γ*. *European Journal of Immunology*.

