# Supplementary material for: Identification of Potential Transcriptional Biomarkers Differently Expressed in Both S. aureus- and E. coli-Induced Sepsis via Integrated Analysis
Source: Biomed Res Int. 2019 Apr 9;2019:2487921. doi: 10.1155/2019/2487921 (PMC6481126; doi:10.1155/2019/2487921)
Supplement: Supplementary Materials — Supplementary Table 1 Primers designed for validation of commonly expressed genes in four datasets by quantitative real-time PCR. Supplementary Table 2 is a summary of DEGs selected from each dataset using |log2 Fold Change| ≥1.5 and FDR < 0.05 as cut-off criteria. Supplementary Table 3 is the common genes in any three datasets. [file 2487921.f1.pdf]

Supplementary Table 1 Primers designed for validation of commonly expressed genes in four dataset by quantitative real-time PCR.

**Primer name**	**Forward sequence (5'-3')**	**Reverse sequence (5'-3')**	**Tm(F/R)**	**Amplicon size**
CEACAM1-human	AACTTTGAAGCCCAGCAACC	GCAGGACAGGTTTCATTACTGC	59.3/59.8	108
PFKFB3-human	AAAGTCCGGAAGCAATGTGC	ATTGGTGGCATCGAAAACCG	59.4/59.5	96
PSTPIP2-human	CTGAAATCAACACCCTGAAGCG	CCTGGCCTCTTCTCTTAAACTC	60.1/58.1	107
SOCS3-human	AGTACGATGCCCCGCTTTAAG	TTGGCTTCTTGTGCTTGTGC	60.5/59.9	107
IL18RAP-human	AGTGAGAACCATTGTGGGAGAC	AGCCAAATCGTGCTTTGCAG	60.0/60.0	119
LILRA5-human	AGGTGTCAGATGTGTCTCTGC	TTTTTCAGCCCTGGAGATGC	59.7/58.5	84
TNFAIP6-human	AAGCACGGTCTGGCAAATAC	ATGGCCGCCTTCAAATTCAC	58.8/59.5	71
GK-human	ACTTCGTCCCAGCATTTTCG	TCCACAGATTATCCCTCTTGCG	58.8/59.9	71
RPS18-human	ATCCTCAGTGAGTTCTCCCG	CTTTGCCATCACTGCCATTA	58.5/56.4	106

Note: CEACAM1: carcinoembryonic antigen related cell adhesion molecule 1; GK: glycerol kinase; PFKFB3: 6-phosphofructo-2-kinase/fructose-2, 6-biphosphatase 3; TNFAIP6: TNF alpha induced protein 6; IL18RAP: interleukin 18 receptor accessory protein; LILRA5: leukocyte immunoglobulin like receptor A5; PSTPIP2: proline-serine-threonine phosphatase interacting protein 2; SOCS3: suppressor of cytokine signaling 3; RPS18: ribosomal protein S18.

Supplementary Table 2 Summary of DEGs selected from each dataset using |log2 Fold Change| ≥1.5 and FDR < 0.05 as cut-off criteria.

**Dataset accession number**	**Upregulated gene in *Staphylococcus aureus*-induced sepsis**	**Downregulated gene in *Staphylococcus aureus*-induced sepsis**	**Upregulated gene in *Escherichia coli*-induced sepsis**	**Downregulated gene in *Escherichia coli*-induced sepsis**
GSE4607	320	177	389	108
GSE25504	132	34	204	156
GSE33341	220	165	154	141
GSE65088	335	116	367	61

Note: FDR: false discovery rate.

Supplementary Table 3 The common genes in any three datasets.

**Upregulated gene in *Staphylococcus aureus*-induced sepsis**	**Downregulated gene in *Staphylococcus aureus*-induced sepsis**	**Upregulated gene in *Escherichia coli*-induced sepsis**	**Downregulated gene in *Escherichia coli*-induced sepsis**
ACSL1	**CCR7**	**ANXA3**	ABLIM1
**ANXA3**	CD247	ARG1	AUTS2
B4GALT5	**CD96**	BASP1	BCL11B
**CEACAM1**	**FCMR**	CA4	**CCR7**
CST7	ID3	CD177	CD52
DRAM1	IL7R	**CEACAM1**	**CD96**
FCGR1B	ITM2A	CLEC5A	FAM102A
**FFAR2**	**LEF1**	**FFAR2**	**FCMR**
**GK**	**LRRN3**	FLOT1	**LEF1**
**GYG1**	LY9	FPR1	**LRRN3**
**HK3**	**NELL2**	**GK**	**NELL2**
**HP**		GPR84	PASK
**IL18R1**		**GYG1**	RASGRP1
IL1RN		**HK3**	
LCN2		**HP**	
**MMP9**		**IL18R1**	
**OLFM4**		IL18RAP	
**PFKFB3**		LILRA5	
**PLSCR1**		LIMK2	
**PSTPIP2**		MMP8	
**RETN**		**MMP9**	
S100A12		**OLFM4**	
**SAMSN1**		ORM1	
**SERPINB1**		PFKFB2	
**SERPING1**		**PFKFB3**	
SLC2A3		PGS1	
**SOCS3**		**PLSCR1**	
**SORT1**		**PSTPIP2**	
**STOM**		**RETN**	
**TNFAIP6**		**SAMSN1**	
TXN		**SERPINB1**	

	**SERPING1**
	SMPDL3A
	**SOCS3**
	**SORT1**
	**STOM**
	TLR5
	**TNFAIP6**
	TNFSF10
	UPP1
	VPS9D1

Note: The bold font represents emerged in both *Staphylococcus aureus* and *Escherichia coli* groups.
